# POSMM: an efficient alignment-free metagenomic profiler that complements alignment-based profiling

**DOI:** 10.1186/s40793-023-00476-y

**Published:** 2023-03-08

**Authors:** David J. Burks, Vaidehi Pusadkar, Rajeev K. Azad

**Affiliations:** 1 Department of Biological Sciences and BioDiscovery Institute, University of North Texas, Denton, TX 76203 USA; 2 Department of Mathematics, University of North Texas, Denton, TX 76203 USA

**Keywords:** Metagenomes, Microbiome, Taxonomic classification, Markov model, Sequence alignment

## Abstract

We present here POSMM (pronounced ‘Possum’), Python-Optimized Standard Markov Model classifier, which is a new incarnation of the Markov model approach to metagenomic sequence analysis. Built on the top of a rapid Markov model based classification algorithm SMM, POSMM reintroduces high sensitivity associated with alignment-free taxonomic classifiers to probe whole genome or metagenome datasets of increasingly prohibitive sizes. Logistic regression models generated and optimized using the Python sklearn library, transform Markov model probabilities to scores suitable for thresholding. Featuring a dynamic database-free approach, models are generated directly from genome fasta files per run, making POSMM a valuable accompaniment to many other programs. By combining POSMM with ultrafast classifiers such as Kraken2, their complementary strengths can be leveraged to produce higher overall accuracy in metagenomic sequence classification than by either as a standalone classifier. POSMM is a user-friendly and highly adaptable tool designed for broad use by the metagenome scientific community.

**Supplementary Information:**

The online version contains supplementary material available at 10.1186/s40793-023-00476-y.

## Background

Shotgun metagenomics is becoming increasingly popular in profiling the taxonomic composition of microbial communities. Wherein the early applications sought to identify the members of microbial communities, further advances in sequencing technologies and analysis tools are uncovering new information that shines a light on hitherto unknown facets of microbiotas and at the same time elicits new questions that call for more inquiries into microbiotas and therefore further interrogation of the metagenomic data. In contrast to 16S based approach where the focus is on sequencing only 16S rRNA genes of a community, shotgun metagenomics strives to sequence the entire nucleotide complement of a microbial community. While the debate remains open on the efficacy of metagenomic profiling through 16S sequencing versus whole metagenome (shotgun) sequencing (WMS) [1, 2], it is beyond question that WMS allows for functional profiling by targeting the entire genomic repertoire of culturable and unculturable organisms in a community.

The increased complexity of WMS datasets demands the development of more advanced methods for taxonomic profiling. Such tools are tasked with determining the taxonomic identities of individual reads arising from taxa that may or may not have representation in the genome databases. Sequence alignment, the standard approach to inferring the origins of nucleotide fragments, can establish the taxonomic identity unambiguously only if the read originating organism is represented in the database. Despite the limitations, alignment algorithms such as BLAST have remained the mainstay in the taxonomic classification of metagenomic reads [3]. Metagenomic classification through local alignment has been augmented by developing extensions of BLAST, such as HS-BLASTN and DIAMOND, which prioritize speed to handle the increasingly cumbersome size of emerging WMS data [4].

Despite the development of ultrafast alternatives to BLAST, the sheer size of metagenomic data has reoriented the focus of alignment towards hyper-fast exact-match for queries of distinct *k*-mers composing the reads [5–7]. In 2017, the typical size of a WMS dataset was estimated between 1 and 10 Gbp [8], and has continued to grow as the associated costs and technological hurdles of sequencing shrink. The advent of third-generation sequencing has further amplified this big data problem in metagenomics [9–12], and the traditional alignment-free approaches adapted for use in metagenomic taxonomic classification are continuously being rendered obsolete despite typically offering higher sensitivity across large phylogenetic breadth [5, 13–15].

Alignment-free methods offer a more robust higher level of taxonomic abstraction for metagenomic sequences compared to methods based on alignment, particularly when the query read originating genome is elusive [14, 16]. Recent years have seen a resurgence of Markov model based methods for metagenomic classification [16–19]. While no current Markovian approach outpaces the optimized alignment schema of tools such as Kraken or CLARK in terms of the turnover rate [5, 6], new optimized algorithms have brought Markov models back as a realistic alternative with reasonable runtimes in the context of WMS analysis [14, 16, 17]. While classification speed is certainly important, accuracy is paramount, and the metagenomic scientific community should have the options to choose one over the other, or perhaps the best trade-off between the two, based on their priorities and needs.

One of the biggest hurdles in taxonomic classification, particularly for reads where the closest identified relative may not share even a single oligomer of reasonable length, is the estimation of confidence for matches. The probabilistic scoring by Markov models does identify the best matching model (genome) to the read but offers little beyond this. Whether the best hit represents the source organism the read originated from is always in question as this does not provide insight into the strength of the relationship between a model and the read. A frequently used Markov model based program, PhymmBL, introduced polynomial functions accounting for read length, Markov model order, and taxonomic level to generate confidence scores in later revisions of the software [20], though the underlying methodology was not clearly laid out. Alignment based program Kraken2 offers a classification score based on the frequencies of taxon-specific *k*-mers, but can vary greatly with the database used, and quickly becomes restrictive, particularly for taxa with highly similar *k*-mer representations [21].

Combining complementary methods has seen success as a strategy to raise the accuracy bar in taxonomic classification. PhymmBL is an example of such an approach that exploited the complementary strengths of interpolated context models (ICMs) generated by GLIMMER [22] and the local alignment with BLAST [23] within an integrative framework to classify reads with higher sensitivity and precision than by either of the standalone programs. Such combinations work best when the strengths of each method can address the weaknesses of the other [24]. For modern classifiers built on exact *k*-mer alignment, precision can be very high. Sensitivity, however, is a usual weakness, with tools such as Kraken failing to align over 68% of reads from real metagenomic datasets [5].

In what follows, we introduce and describe a new metagenomic classifier, POSMM (pronounced ‘Possum’), named after Python-Optimized Standard Markov Model algorithm. POSMM leverages higher accuracy of alignment-free, Markov model based approach for taxonomic abstraction as both a standalone program and a component program for WMS read classification. Building Markov models of genomes and scoring of reads by the trained models are executed by our previously published standard Markov model (SMM) based algorithm [16], allowing the end-user to select the model order and therefore control the accuracy and CPU time trade-off (computationally demanding higher order models tend to be more accurate, however, this may not be always true). The taxonomic classification of reads is performed based on a regression-based probability score derived from simulated read data. The training dataset was assembled by proportionately sampling from genomic regions with distinct compositional signatures for each prokaryotic species represented in the database; precautions were taken to remove the contaminant sequences that may distort the conclusions of our machine-learning process. This was achieved by employing the Segmented Genome Model (SGM) based program, a new C++ incarnation of an integrated segmentation and clustering program that can rapidly segment genomes and group compositionally similar segments into distinct clusters for each genome [25–27].

## Methods

### Database generation

Developing a fully inclusive database is essential for training and testing any taxonomic classification method. Only including the highest quality genomes can give uncharacteristic advantages during benchmarks that may not be reflected in real-world applications. While Kraken2 maintains a robust standard database and a prokaryotic database, many of the genomes in the mock shotgun dataset [28] and identified in the real metagenomes were not present in either.

POSMM’s speed is dependent upon the number of models (i.e. genomes) being queried. To keep analysis within a reasonable time window and give all species with sequenced genomes equal representation without redundancy, we developed a priority system for collecting representative genomes for all species currently available in NCBI GenBank. First, the archaeal and bacterial assembly summaries were downloaded from the RefSeq release FTP site (https://ftp.ncbi.nlm.nih.gov/refseq/release/). Taxid numbers were used to isolate unique species and then a representative genome for each species was obtained. Using the NCBI RefSeq terminology, included with the assembly summary, we selected ‘Reference’ genomes where available, otherwise ‘Representative’ genomes. When neither of Reference and Representative genome was available, the decision was based on the assembly level in the order of ‘complete’, ‘chromosome’, ‘scaffold’, and finally ‘contig’. Species with only a partial representation of their genomes were not included in our custom database. In the event of a tie, one genome was randomly chosen using a random number generator from the Python standard library.

The custom database is comprised of genomes of 29,870 unique species (Additional file 1: Table 1). These genomes represent various quality levels; partial genome assemblies were not included. Because of variable quality of the genome assemblies, each genome was subjected to filtering for potentially extraneous sequences. The same type of genome set can be downloaded using the POSMM “–runmode setup” and “–gtype bacteria/archaea” parameters.

Furthermore, two additional recently developed programs KrakenUniq [29] and Kaiju [11] were assessed against Kraken2, POSMM, and the hybrid of Kraken2 and POSMM, on the simulated metagenomes. For KrakenUniq, a custom database of bacterial and archaeal genomes was built. For Kaiju, the NCBI RefSeq database was used.

### Real and mock metagenome processing

WGS reads from male human saliva samples (NCBI SRA accessions SRR062462 and SRR062415) were downloaded using the fastq-dump utility from the sratools suite [30]. Reads were trimmed to remove low-quality bases and adapter sequences using the fastp program [31]. Human DNA sequences were removed by aligning reads to the *Homo sapiens* genome using the BWA program [32]. Specifically, reads from each dataset were first trimmed using fastp 0.20.1 at the default setting and were then aligned onto the human reference genome (build GRC38h, GenBank assembly accession: GCA_000001405.15) using bwa 0.7.17-r1188 at its default setting and “bwa mem” mode. The unmapped read IDs from the output SAM/BAM files were parsed using samtools (view) 1.11 and were used in pre-processing of the original FASTA/FASTQ files, and thus removing the human-mapped reads in the process. Sunburst diagrams for taxonomic classifications were generated using the plotly library for Python 3.8.

NCBI SRA accession SRR8073716, representing an Illumina-sequenced metagenome from a previously published mock microbial community [28], was also downloaded via fastq-dump. No read processing was performed prior to analysis by either program (Kraken2, POSMM). Direct genome read alignment counts were taken from the supplementary files of the original study [28].

### Markov model classification algorithm

POSMM allows users to build standard Markov models or SMMs of orders 10–12 [16] for each genome using the genomic sequence fasta files. First, an empty count distribution of the specified order is filled with pseudocounts. At the start of each run, an “empty” probability distribution is also built, representing the initial and transition probabilities for the specified model order *k*. Both initial and transition distributions are kept global and are reset as each genome is modeled. In this way, metagenomic fasta files can be indexed respective to the relevant positions of the global probability distribution by using a vector of memory-address pointers. Maintaining a static location in memory and changing the probabilities per genome minimize the memory footprint and avoid I/O bottlenecks. The average model build time for a prokaryotic genome is typically less than 3 s, but can be further minimized by storing genomic data on high-speed NVMe or RAMDisk drives.

To speed up the throughput, POSMM splits genome sets for modelling based on the specified CPU core availability, and runs concurrent analyses of the same metagenomic fasta file. This is faster than multi-threading the reads being analyzed, and takes full advantage of the increasing RAM availability of the modern computing environments. The biggest bottleneck of SMM, and by association, of POSMM, is on-the-fly generation of Markov models of genomes, however, splitting this across multiple CPU cores bestows the highest performance gains to the user.

### Machine learning derived score

The motivation for this analysis is conversion of raw model probabilistic scores into threshold-based values, in order for users to set cutoffs for classification. Note that all reads are assigned scores using the model used. Deciphering if those scores are useful in the context of this study required additional insights, and we have attempted here the use of machine learning (regression) to provide this.

Most alignment-free metagenomic classifiers tend to assign taxonomic identity to all reads regardless of whether the source taxa for the reads are represented or not in the genome database used for classification [14, 33]. This can lead to an inflation of misclassifications, particularly for reads originating from organisms whose genomes are not represented in the genomic databases. Higher taxonomic level classification could be more accurate as closely related genomes belonging to the same taxon may be represented in the database; however, even higher level classifications are not immune to this as a vast number of reads in a metagenomic sample may not have their source representation even at higher taxonomic levels in the database. Alignment based methods have largely avoided this problem as alignment provides a confidence score for the similarity of the query read with a subject sequence in the database. This may reduce misclassifications resulting from ambiguous alignments [5, 6, 21]. The developers of alignment-free classifier PhymmBL took a cue and attempted to address by introducing a confidence score akin to the alignment score [20]. Using simulated training data, 3D-curve fitting was applied in order to formulate a similarity score based on the read length, taxonomic level, and Phymm score. Thresholding based on this score was demonstrated to be effective in reducing misclassifications [5]. However, later studies have suggested that using this score for thresholding can lower both sensitivity and specificity of metagenomic classification [34].

The fidelity of any fitting procedure is dependent upon the quality of the training data. Poor taxonomic representation, or perhaps taxonomic overrepresentation, could explain why certain datasets seem to benefit the scoring schema of PhymmBL while others do not [5, 34]. To develop a more robust Markov model based scoring schema for phylogenetic classification, we employed logistic regression in combination with Bayesian optimization and cross-validation techniques. Furthermore, training data was sampled from the compositionally distinct fractions within each genome [27]. Representation of compositionally disparate regions within genomes is vital for producing a reliable score. Attempts to generate higher order models of compositionally atypical regions did not yield desired results as these regions were often relatively much small and therefore did not lend themselves well to generating reliable higher order models. The increase in the number of models also dramatically increased the POSMM’s runtime. Isolating these regions, and having their representation in the training data, was deemed an effective approach for incorporating useful evolutionary information encoded within prokaryotic genomes.

### Simulated training set construction

Training sets were necessary to properly employ regression in a way that converted the raw model scores to relative, bound values. Contaminations in GenBank genome assemblies are a documented problem. Contaminant sources, such as extraneous DNA or adapter sequences, must be identified and eliminated. On the other hand, horizontally acquired genomic regions are commonly present across prokaryotes and are integral parts of their genomes [26, 27, 35]. Not adequately accounting for these mobile elements in genomes could result in the misclassification of a significant fraction of metagenomic reads.

In addition to horizontal gene transfer, genomic mosaicism may arise due to other evolutionary or biological factors [27]. These compositionally disparate regions need to be accounted for in order to render a genome model that adequately represents the variability within a genome. For example, there must be distinct models representing horizontally acquired regions from distinct lineages and a model representing the vertically transmitted regions in a genome. Accounting for mosaic compositional structure of prokaryotic genomes is paramount to establishing a high-quality training dataset for regression. To address this, we used the Markovian Jensen–Shannon divergence (MJSD) based segmentation and clustering method that has previously been applied to predict genomic islands in prokaryotic genomes [25, 27]. This enabled isolation of compositionally distinct regions within each genome in our custom genome database. An optimized algorithm, based on the same methodology for segmentation and clustering as in IslandCafe [27] but designed to be computationally more efficient, allowed analysis of genomes at a rate capable of handling the entire RefSeq database on a single desktop computer within a reasonable time. For our test system based on a Ryzen 1600 CPU, our segmentation and clustering algorithm processes approximately 2 prokaryotic genomes per minute using all 6 physical cores. The new algorithm uses an optimized technique for computing entropies to estimate the divergence between DNA sequences through MJSD. The new algorithm uses a reverse-calculation step that allows rapid nucleotide-wise iteration across the entire genome (see below). This resulted in a 16-fold reduction of the average time for segmentation and clustering of a prokaryotic genome (average size ~ 5 Mbp), from over 41 min to approximately 2.5 min. For segmentation, we recursively iterated divergence computation at each position of the genome and segmented at the position with the highest MJSD between two resulting subsegments provided the associated p-value was less than 0.05. The significance threshold for clustering was set to 10^−5^ (readers should refer to Azad and Li [25] or Jani and Azad [27] for details).

Clusters less than 0.001% the size of the genome were discarded. The remaining clusters were queried for human, viral, and adapter sequence contamination using BLAST and those with significant similarity to these were also discarded. As segments within a cluster are compositionally similar, we expect these segments to generate more similar Markov model scores than the segments from different clusters. By using a random number generator, we generated fragments of random lengths between 30 and 500 bp from each cluster to generate labeled fragment sampling pools. Randomly sampling fragments from each cluster ensured representation of each compositionally distinct region in our training data. Multiple datasets of 250,000 reads were randomly sampled from these pools to generate 10 unique metagenomic training datasets for each taxon (phylum, class, order, family, genus, and species). By cycling through these datasets with a Bayesian optimization scheme (see below), we generated regression models that were used for taxonomic classification of reads as further discussed below.

### Markovian Jensen–Shannon Divergence (MJSD) based segmentation and clustering algorithm

In building the training set, we used an advanced method based on Markovian Jensen–Shannon divergence (MJSD) to obtain the core (native) components of all available prokaryotic genomes to ensure the most balanced representation was used in our regression. We were able to significantly reduce the runtime of genome segmentation and clustering algorithm, as implemented in IslandCafe [27], by introducing a reverse-calculation step during recursive segmentation. MJSD, entropy, and statistical significance were calculated as described in [27]. Specifically, the information content of a genome sequence, quantified by the entropy function for probability distribution *p*_*i*_, is obtained as, $H^{m} \left( {p_{i} } \right) = - \mathop \sum \limits_{w} P\left( w \right)\mathop \sum \limits_{{x \in {\mathcal{A}}}} P(x|w)\log_{2} P(x|w)$, where *P*(*x*|*w*) is the probability of nucleotide *x* given the preceding oligonucleotide *w* of length *m* (*m* defines the model order, is set to 2 in IslandCafe) and *P*(*w*) is the probability of oligonucleotide *w*. A genome is initially segmented by iterating the computation of entropy and thus MJSD at each position along the genome and identifying the location of highest MJSD of (user-defined) significance in the genome. This process is then iterated for the resulting genomic segments.

### Augmenting computational efficiency of segmentation and clustering algorithm

IslandCafe reduces the runtime by computing MJSD at every *l*/10000th position along the genome sequence of size *l* to be segmented, however, it computes afresh the probability parameters using the oligonucleotide counts for each MJSD computation. In contrast, we designed our new segmentation and clustering algorithm to iteratively computes MJSD at each nucleotide position in the genome. However, for each subsequent MJSD computation, rather than estimating the entropies afresh, the entropy values from the previous computation were adjusted based on only the oligonucleotides that have to be included and excluded in the current computation. This new approach is not only faster (e.g. over 16X faster than the current segmentation and clustering program on an *E. coli* genome of size ~ 4.7 Mbp) but also results in higher precision as MJSD computation is performed at each position, rather than *n*th position, in the genome.

### Machine learning derived score models

To establish cutoffs based on probabilistic scores, we applied machine learning libraries to the raw SMM scores [16] of our sampled genomic fragments. We used Bayesian optimization to assign hyper-parameters for the logistic regression estimators of the scikit-learn library [36] using the skopt BayesSearchCV module (https://scikit-optimize.github.io/stable/modules/generated/skopt.BayesSearchCV.html). We also tested SVM estimators with a linear kernel, however, the accuracy was on average lower than the logistic regression accuracy for all taxa, and the SVM estimators were found to be prone to overfitting.

Training data containing raw scores outputted by SMM, read lengths, and classification accuracy (True/False) from the top 50 scores for multiple 250,000 read simulated datasets were obtained following SMM analysis at 10th, 11th, and 12th order. We focused on the top 50 scores for each read of our simulated data, as this maintained a balance between the number of correct and incorrect classifications for our regression analysis. Model order and taxonomic rank specific training datasets were obtained, and individual regression models were optimized for phylum, class, order, family, genus, and species levels at 10th, 11th, and 12th model orders. Additional variables, such as read %GC, model %GC, and read entropy, were tested as potential training features, but they added unnecessary overhead with no appreciable gain in classification accuracy following the model training.

The scikit-optimize BayesSearchCV function allows for parameter optimization and model fitting using a “fit” and “score” method. A threefold cross-validation was performed; the training data was randomly split into 3 groups during each optimization test. The first two sets were used for model training and validation respectively at various parameter combinations and the third set was used for testing the trained regression model. Performance was assessed by applying the trained regression models to the test data and determining the classification accuracy. Unlike grid search optimization, which tests all possible combinations of hyper-parameters, Bayesian optimization adjusts hyper-parameters based on prior performance results. Users are required to set static values or ranges for the model being optimized. We used the sklearn.linear_model.LogisticRegression module of scikit-learn as our model generator, and kept settings for dual formulation and 15,000 iterations constant for each training session. Otherwise with dual = false and lower iteration values, the logistic regression classifier may fail to converge. The inverse regularization parameter, referred to as the C parameter in scikit-learn, was sampled at values ranging from 1e−6 to 1e5. The tolerance value parameter was sampled with values ranging from 1e−7 to 1e−2. The L1 and L2 penalty norms, ‘liblinear’ and ‘saga’ solvers, and intercept fitting booleans were cross compared for various combinations by the BayesSearchCV function. Optimized parameters are included in (Additional file 1: Table 2), and guided final model building. All regression models were exported and stored in JSON format using the sklearn–json library.

While these models confer the ability to predict taxonomic identity, the predict_proba function of the final models provides probabilistic score (value in range 0–1) for thresholding, allowing users to prioritize precision over sensitivity at increasing stringencies.

### Sensitivity, precision, and score calculation

Sensitivity and precision were calculated as described in Kraken’s and CLARK’s benchmark studies [5, 6]. In some cases, a genome may not have a taxonomic label for all ranks (species, genus, family, etc.); previous benchmarks have established taxon level accuracy, e.g. genus-level sensitivity is computed as *A*/*B* where *A* is the number of reads with the genera correctly assigned by a method and *B* is the total number of reads of known genera. Sensitivity was calculated similarly for all other taxonomic ranks.

Precision is also based on the definition established by prior benchmarks, wherein the genus-level precision is calculated as *X*/(*X* + *Z*), where *X* is the number of reads with genera correctly assigned by a method, and *Z* is the number of reads with an incorrect genus assignment by the method. As with sensitivity, precision was calculated independently for each taxonomic rank.

Kraken2’s confidence thresholds were implemented using the—confidence parameter. Thresholds of 0.25, 0.50, and 0.75 were each tested. When the confidence threshold option is invoked, Kraken2 classifies a read to the lowest taxonomic rank satisfying that confidence score.

POSMM’s scores are based on the predict_proba function of scikit’s logistic regression models. The POSMM score for a read to be assigned to a taxon is therefore the probability that the read with the specified score, length, and model order would be assigned to that taxon based on the logistic regression model for that taxonomic rank. Each taxonomic rank and each model order have their own regression models, and probabilistic scores are calculated independently for each.

### POSMM software release

The underlying algorithm for POSMM is written in C++, with all user interface and downstream processing written in Python. Source code for generating probabilistic scores using logistic regression models, written in JSON, as well as all other source codes, are available at https://www.github.com/djburks/POSMM. Simulated metagenomes are available at the Kraken2 website https://ccb.jhu.edu/software/kraken/dl/accuracy.tgz, while the mock and real metagenomes are available at the NCBI SRA [30].

A Python source distribution is also available at https://github.com/djburks/POSMM/blob/main/dist/POSMM-1.0.tar.gz, which handles all necessary dependencies for the end user when installed with pip. Genomes for modeling can be provided by the user with a custom lineage map, or downloaded using POSMM’s internal RefSeq query system by using the—taxlist parameter and a list of GCF numbers.

## Results

Underlying POSMM is a modified version of the original SMM algorithm previously found superior in both classification accuracy and computational performance to legacy Markov model variants [16]. The SMM algorithm was used to build higher order Markov models (order 10–12) of each genome. Each read from a metagenomic dataset is then “matched” against the genome models by computing the probability of the read to be generated by each model. Thresholds for predicting the lineage (different taxonomic ranks) were established based on taxon-specific logistic regression models (see “Methods” Section). First, POSMM was benchmarked against Kraken2, and also against recently published classifiers KrakenUniq [29], and Kaiju [11] on simulated metagenomic datasets. Next, the best performing classifiers were employed to characterize real metagenomic datasets. Furthermore, a combined framework of alignment-free (POSMM) and alignment-based (Kraken2) methods was benchmarked on the same datasets, as described below.

### Real, mock, and simulated metagenomes for classification accuracy assessment

To benchmark the performance of alignment-free POSMM relative to alignment-based Kraken2, KrakenUniq, and Kaiju, we used the simulated metagenomic test datasets as used in both the Kraken and CLARK benchmarks [5, 6]. The HiSeq and MiSeq datasets represent artificial metagenomes assembled from real whole-genome shotgun datasets, whilst the simBA-5 dataset features bacterial and archaeal reads with 5X the error rate expected in metagenomic sequencing [5, 6].

A predefined mock metagenome, developed as part of a study comparing metagenomic sequencing methods [28], was also used to compare the performance of Kraken2 and POSMM, as well as a hybrid of both the programs. Developed from Illumina sequencing of a synthetic microbial community, the full dataset consists of over 213 million paired-end 151 bp reads. The size of this dataset makes it computationally prohibitive for alignment-free classification methods, such as NBC and PhymmBL [5]. Only one of the reads of each pair was used for classification. Comparison was also made to directed alignment performed in the original study using the bwa aligner and the reference genome of each species in the synthetic community [28, 32].

We also analyzed two real human saliva metagenomes that were earlier used in the Kraken and CLARK benchmarks [5, 6]. As with the simulated and mock metagenomes, a significant proportion (over 20%) of reads within these datasets were not classified by Kraken2. Using the custom GenBank database, we classified reads in each dataset with Kraken2 and POSMM. Reads that could not get classified by Kraken2 were re-analyzed with POSMM to assign taxonomic identities to reads otherwise deemed ‘unclassifiable’.

### Establishing score cutoffs for classification

**Fig. 1:**
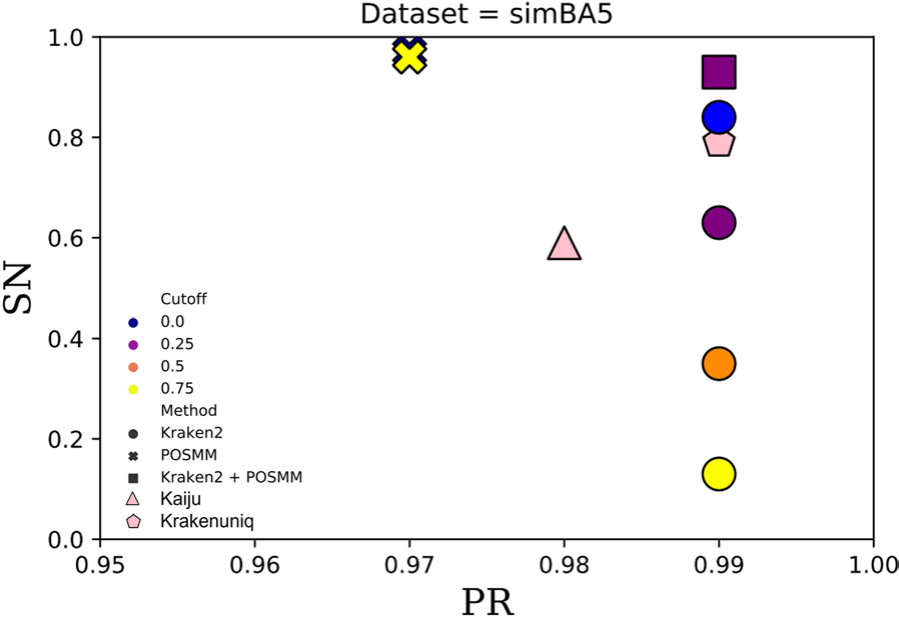
Scatterplot of the genus-level sensitivity (SN) and precision (PR) for Kaiju, KrakenUniq, POSMM, Kraken2, and a hybrid of Kraken2 and POSMM assessed on the simBA5 simulated metagenome dataset. For the hybrid of Kraken2 and POSMM, score cutoffs of 0.0 and 0.25 were used, respectively

**Fig. 2:**
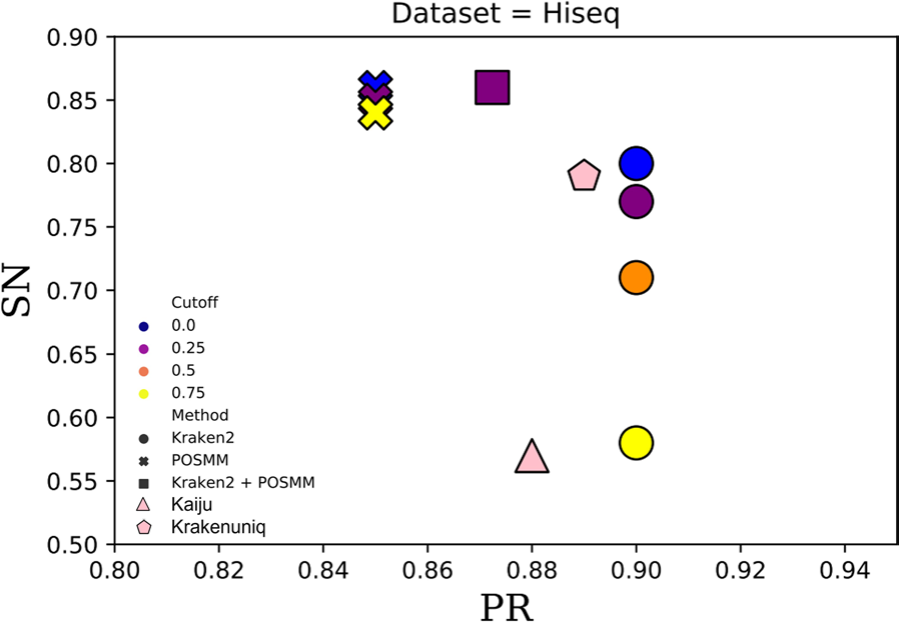
Scatterplot of the genus-level sensitivity (SN) and precision (PR) for Kaiju, KrakenUniq, POSMM, Kraken2, and a hybrid of Kraken2 and POSMM assessed on the HiSeq simulated metagenome dataset. For the hybrid of Kraken2 and POSMM, score cutoffs of 0.0 and 0.25 were used, respectively

**Fig. 3:**
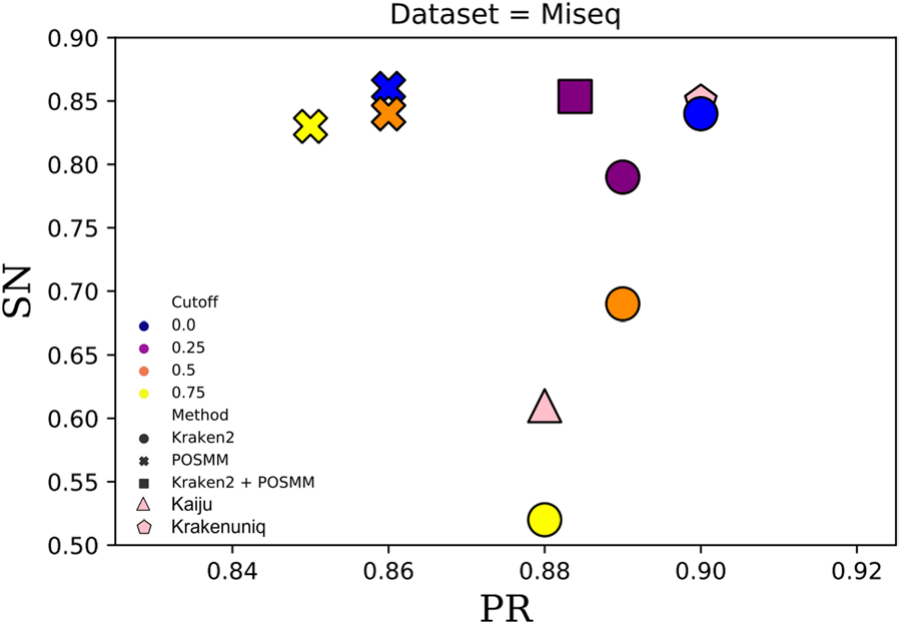
Scatterplot of the genus-level sensitivity (SN) and precision (PR) for Kaiju, KrakenUniq, POSMM, Kraken2, and a hybrid of Kraken2 and POSMM assessed on the MiSeq simulated metagenome dataset. For the hybrid of Kraken2 and POSMM, score cutoffs of 0.0 and 0.25 were used, respectively

POSMM’s thresholding is based on probabilistic scores produced by logistic regression models. To evaluate the effects of score cutoffs on classification precision and sensitivity, reads of each simulated metagenome were classified at different cutoffs, ranging from 0 to 0.75 (Figs. 1, 2 and 3). On the other hand, Kraken2 provides confidence scores for thresholding, however, Kaiju and KrakenUniq do not and were, therefore, compared with the other tools at their default settings. In all cases, sensitivity and precision were calculated as established in the Kraken and CLARK studies (see “Methods” Section) [5, 6].

For mock and real metagenome analysis, the confidence score threshold was not used with Kraken2. For genus-level classification of reads of the simulated and mock metagenomes, Kraken2 performed best without any confidence thresholds (default setting that allows classification to lowest common ancestor based on the number of exact *k*-mer matches in a clade). POSMM performed best with cutoffs ranging typically between 0 and 0.2. As expected, higher cutoffs increased the precision of POSMM at the expense of sensitivity. For the hybrid of Kraken2 and POSMM, a cutoff of 0.25 was used for POSMM (default, performance at other cutoffs are shown in Figs. 1, 2 and 3). When analyzing reads from closely related species, we observed that the 0.25 cutoff did not offer any advantage over no cutoff where the taxon assignment was based on the highest scoring genome model. However, when the dataset contains distantly related reads, beyond phyla, the use of cutoff was observed to improve the classification. In general, POSMM emphasized sensitivity over precision, whereas Kraken2 emphasized precision over sensitivity. After performing an initial analysis with Kraken2, POSMM can be deployed to classify reads that are left unclassified or to provide more specific classifications to reads assigned only to higher taxa by Kraken2. This approach leverages the complementarity of Kraken and POSMM, that is, the speed and precision of Kraken and the sensitivity and capability to classify at different taxonomic ranks of POSMM.

### Simulated metagenome classification accuracy

To assess the classification performance of the previously described tools we used simulated, mock, and real metagenomes. Simulated and mock metagenomes allow for performance reporting, as the read identities are pre-established (simulated metagenomes) or narrowed to known members of the originating synthetic microbial community (mock metagenomes). Real metagenomes offer additional insights into the real-world applicability of POSMM and Kraken2, as well as the benefits of combining both approaches, but offer little in terms of the accuracy of either method.

For a fair assessment, we used the previously established test metagenomes, namely, the simulated metagenomes featured in Kraken and CLARK’s original benchmarks [5, 6], as well as in other classifier-performance studies [37, 38]. For simulated metagenomes, the precision and sensitivity were computed at different score thresholds as well as without a threshold.

The precision of Kraken2 was high, close to 0.9 or greater, for all three simulated metagenomes (Figs. 1, 2 and 3). The lowest precision reported by Kraken2, 0.878, was observed with the MiSeq metagenome at the cutoff of 0.75. However, this is just 0.02 less than the highest precision of 0.898 reported by Kraken2 for this dataset at no confidence score cutoff. Kraken2 generated lower sensitivity overall in comparison to the other tools, which declines sharply as the score cutoff was increased (Figs. 1, 2 and 3). This was due to increase in the number of “unclassified” reads as the threshold was increased. POSMM, on the other hand, showed higher sensitivity in classifying reads in comparison to the other tools (Figs. 1, 2 and 3). POSMM’s precision was slightly lower on the simulated datasets, however, it did not drop below 0.851, which was observed with the HiSeq dataset at the cutoff of 0.75. This was just 0. 042 less than the precision of Kraken2 on the same dataset.

**Fig. 4:**
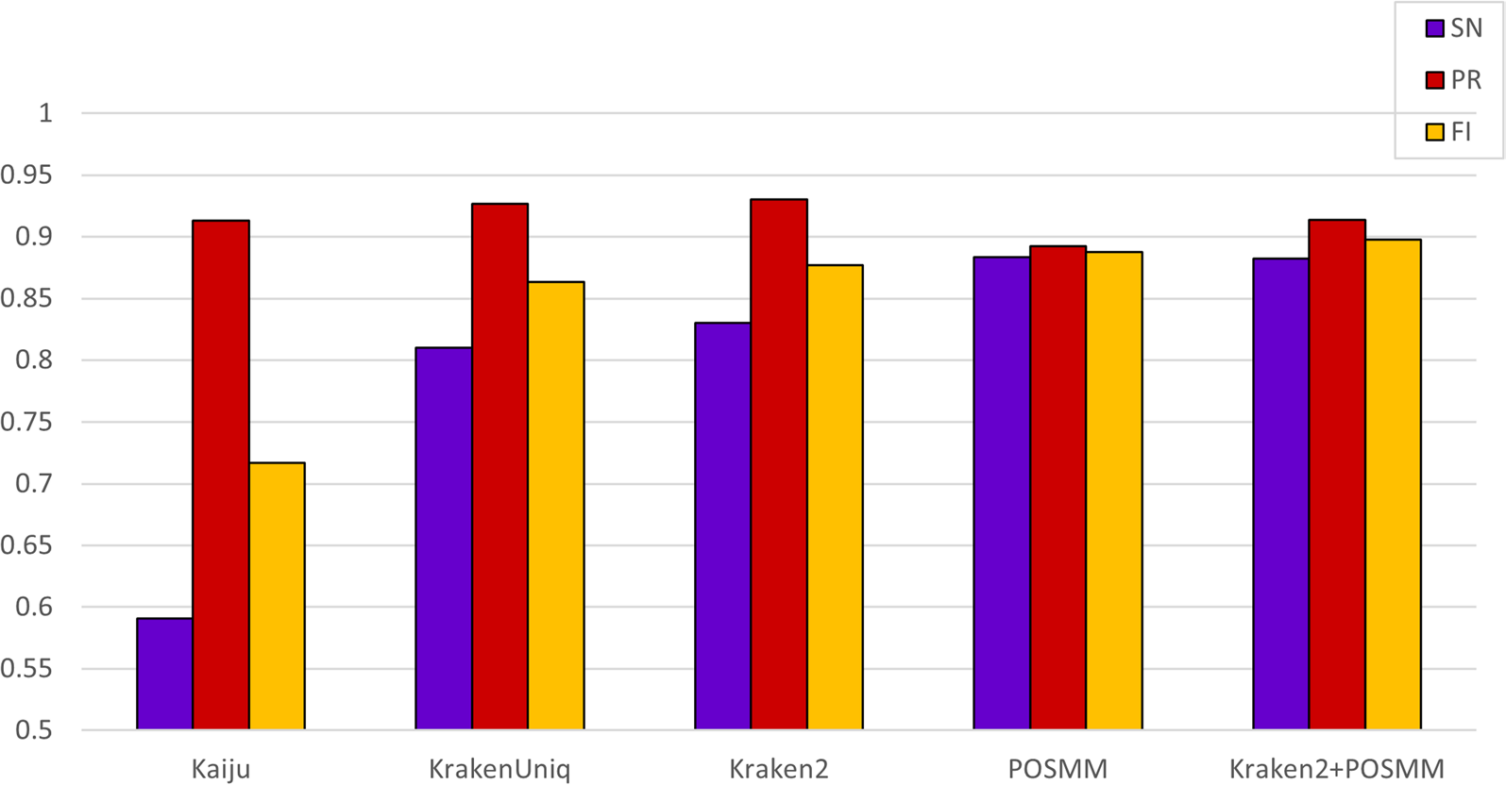
Genus-level performance scores, namely, sensitivity (SN), precision (PR), and F1-score, averaged over three simulated metagenomes (Hiseq, Miseq, and simBA-5) for Kaiju, KrakenUniq, POSMM, Kraken2, and a hybrid of Kraken2 and POSMM. Kraken2 was assessed without a confidence threshold applied. A score cutoff of 0.25 was used for POSMM. For the hybrid of Kraken2 and POSMM, initial classification was obtained with Kraken2 without a cutoff, followed by genus level classification of reads left unclassified by Kraken 2 with POSMM at 0.25 cutoff

Increasing the threshold above 0.25 did not result in any noticeable improvement in POSMM’s performance; in contrast, this results in significant decline in the sensitivity of Kraken2. We, therefore, created a hybrid of POSMM and Kraken2 at threshold for POSMM set to 0.25 and threshold for Kraken2 set to 0. The POSMM-Kraken2 hybrid yielded the best overall performance (highest F1-score, the harmonic mean of precision and sensitivity) on the simulated metagenomes (Fig. 4). By first analyzing each simulated metagenome with Kraken2, and then applying POSMM only to reads left unclassified by Kraken2, the highest F1-scores for all three simulated metagenomes could be achieved (Additional file 2: Figure 1). The most pronounced improvement in performance was observed with the SimBA5 dataset, where the hybrid approach yielded an F1-score 4.4% higher than the next best performing method, Kraken2 (Additional file 2: Figure 1a). The effect was less obvious with the HiSeq and MiSeq datasets (~ 1% change in F1-score), but only when compared to Kraken2 without any confidence score thresholding (Figs. 2 and 3). The increase in F1-score attained through the hybrid approach is mainly due to an increase in sensitivity contributed by POSMM. The hybrid approach produced the highest mean sensitivity (0.883) and mean F1-score (0.898). The average precision of the hybrid approach was slightly lower than that of Kraken2 (by 1.6%) but was offset by a gain in sensitivity through POSMM resulting in superior overall performance (Fig. 4).

The overall accuracy (F1-score), averaged over the three simulated metagenomes (Hiseq, Miseq, and simBA-5), for the hybrid tool was 0.898, while it was 0.877 for Kraken2. Note this improvement of 2.1% in the overall accuracy resulted in over 1,500 more of metagenomic reads (of total 30,000) classified correctly. Of the total 30,000 metagenomic reads from the three simulated metagenomes, Kraken2 correctly classified 24,913 (83.04%) at the cost of 1923 incorrect classifications (6.41%) and 3,164 no classifications (10.55%) (that is, 16.96% incorrectly classified or unclassified). In contrast, the hybrid tool correctly classified 26,476 (88.25%) at the cost of 2521 incorrect classifications (8.4%) and 1,003 no classifications (3.34%) (that is, 11.74% incorrectly classified or unclassified). That is, 1563 more reads (5.21%) were classified correctly by the hybrid method; perhaps this was achieved by classifying correctly a large number of reads left unclassified by Kraken2 (a decrease to 3.34% from 10.55%), with disproportionately a small increase in incorrect classification (8.4% from 6.41%). Our results highlight the complementary strengths of POSMM (sensitivity) and Kraken2 (specificity) that can be leveraged to raise the accuracy bar in metagenomic classification.

KrakenUniq was slightly outperformed by Kraken2. Kaiju, although found precise, produced very low sensitivity values on the simulated metagenomes (Fig. 4). Consequently, KrakenUniq and Kaiju generated lower mean F1-scores of 0.863 and 0.716 respectively. Therefore, these tools were not considered in the further downstream analysis.

As expected, alignment-based methods perform well in “unmasked” scenarios, when the exact counterparts of metagenomic sequences are available in the database (with some variations or mutations allowed), and thus could be the tools of choice for species level classification in such scenarios. Kraken2, for example, compares favorably in the species level classification (Additional file 1: Table 3). However, as only a very small fraction of microbial communities (~ 1–10%) are represented in the databases, the performance of alignment-based methods can decline remarkably in the classification of reads whose originating taxa are not represented in the database. To circumvent this problem, we recommend the Kraken2-POSMM hybrid approach, wherein Kraken2 can provide the species-level classification, and for those reads that cannot be classified by Kraken2 with the desired confidence level (due to a lack of representation in the reference database), POSMM can aid in higher level of taxonomic classifications. This was demonstrated by our analysis of these tools and their hybrid using simulated datasets as discussed above.

### Mock metagenome classification accuracy

**Fig. 5:**
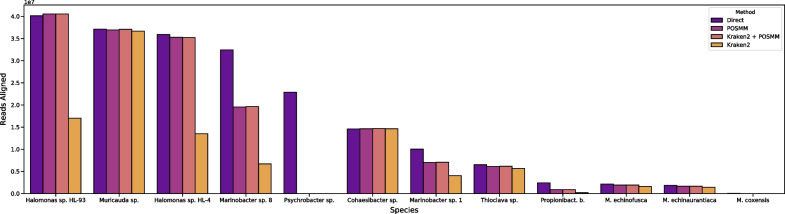
The number of reads assigned by POSMM at 0.25 cutoff and Kraken2 (no cut-off) to each species represented in the SRR8073716 mock metagenome. For the hybrid of Kraken2 and POSMM, initial assignment was obtained with Kraken2 without a cutoff, followed by genus level assignment of reads left unassigned by Kraken 2 with POSMM at 0.25 cutoff. “Direct” refers to read assignment using genome-specified bwa alignments in the original study

Next, the performance of POSMM. Kraken2, and their hybrid was assessed on the mock metagenome and also compared with the genome-directed alignments performed in the original study of the mock metagenome (Fig. 5) [28]. No read filtering was performed prior to analysis with POSMM or Kraken2. Similar to the simulated metagenome results, Kraken2 left millions of reads unclassified at several taxonomic ranks as confidence thresholds were introduced. POSMM, as before, helped classify reads that were deemed ‘unclassifiable’ by Kaken2 or even by the original study [28].

**Fig. 6:**
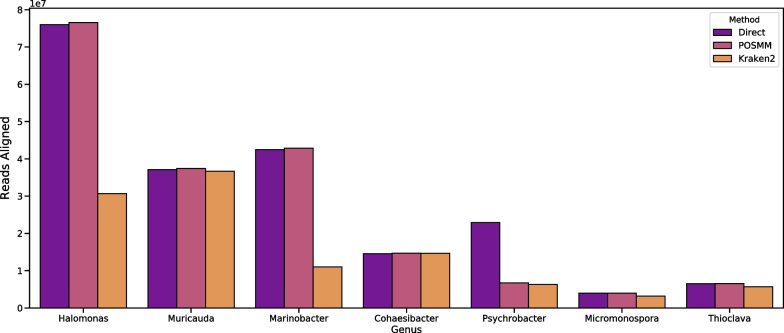
Same in Fig. 3 but for genus level. Genera represented in the mock metagenome are shown

Kraken2 did not do well in assigning reads belonging to the *Halomonas* genus, which constituted the ~ 37% of the mock community, specifically in the assignment of the reads to either of the two *Halomonas* species (HL-93, HL-4), or to the genus itself. On the other hand, POSMM’s read assignment matched closely with the genome-directed bwa alignments in the original study. The *Psychrobacter* species of the mock community (LV10R520-6) was not represented in the genome database used for Kraken2 and POSMM, and as expected, reads from this species were misclassified at the species level. Genus level classification was also not up to the mark (Fig. 6), despite the inclusion of 58 unique *Psychrobacter* species in the database. POSMM aligned more reads specifically to the two *Marinobacter* species genomes (LV10MA510-1 and LV10R510-8), and this was also reflected at the genus level, where POSMM shared more read alignment to these taxa with the direct-alignment (~ 42 million reads) than Kraken2 with the direct-alignment (~ 11 million reads).

Interestingly, combining POSMM and Kraken2 very closely resembled the results of POSMM standalone (Fig. 5). As before, the entire mock metagenome was first analyzed with Kraken2 without a threshold. Reads that were not assigned to any species by Kraken2 were then reanalyzed by POSMM with a 0.25 score cutoff, and then the taxonomic classifications were merged. Given the size of the dataset, this led to a dramatic decrease in POSMM analysis time, as Kraken’s first-pass analysis filtered out over 51% (over 109 million reads) of the dataset.

### Real metagenome classification comparison

We used both Kraken2 and POSMM to characterize the communities of two human microbiome samples previously featured in multiple metagenomic classification benchmarks [5, 6]. Unlike previous assessments, our full GenBank database was used for read classification by both Kraken2 and POSMM. Datasets SRR062462 and SRR062415 are both of human saliva samples and were filtered for human contaminant reads prior to the analysis. Quality-trimming and additional filtering were performed, removing low quality bases and adapter remnants.

The proportions of genus classifications were similar between Kraken2 and POSMM (Additional file 2: Figures 2–7). As with the simulated and mock metagenomic datasets, Kraken2 left a significant number of reads unclassified (> 277,000 reads, > 20%), which were assigned by POSMM. Despite the difference in total read assignments, the proportions of taxon assignments were similar between these classifiers. In agreement with the prior analysis [5], *Streptococcus*, *Haemophilus*, and *Prevotella* genera represented the majority of reads for both programs (Additional file 2: Figures 2–7).

To investigate the reads left unclassified by Kraken2, we filtered reads that were not assigned to any taxon by Kraken2. These reads were subjected to classification by the hybrid of Kraken2 and POSMM, at POSMM cutoff of 0.25. Taxonomic classification of the formerly unclassified reads was fairly spread out across multiple genera. *Bacillus*, the genus with least number reads assigned to it by Kraken2, had now 12,413 additional reads assigned to it by the hybrid program (4.34% of all unclassified reads in the SRR062415 dataset). *Streptomyces* that had only 233 reads assigned to it by Kraken2, was assigned 9745 additional reads by the hybrid program (3.41% of all unclassified reads). Interactive diagrams of the classifications by each method, as well as the POSMM classification of Kraken2’s unclassified reads, built using Plotly and compatible with modern internet browsers, are provided as supplementary html diagrams (Additional file 2: Figures 2A–7A).

### POSMM runtime

**Fig. 7:**
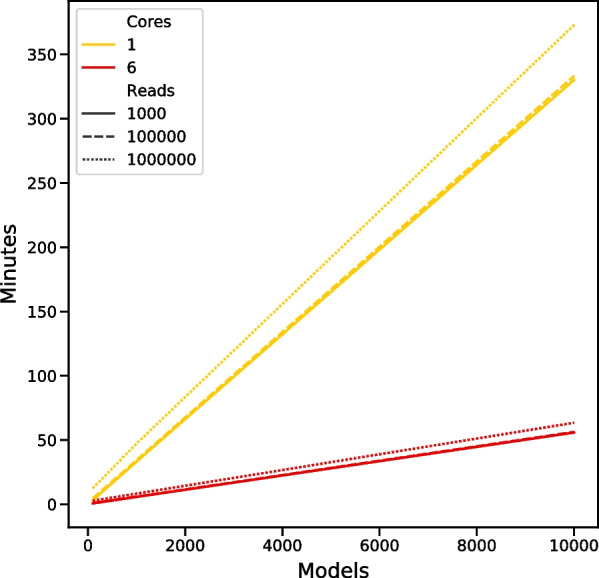
Runtime (in minutes) of POSMM as a function of number of models, number of reads to analyze (size 100 nt), and core count

POSMM runtime is dependent on model order and the number of models used to score the metagenomic reads. We examined POSMM’s time to completion on a single core versus all 6 physical cores in the use of a Ryzen 1600 system. The runtime as a function of dataset size, number of models used, and threading is shown in (Fig. 7). Dataset size (in reads) had little effect on POSMM’s total runtime.

## Discussion and conclusions

POSMM echoes the higher sensitivity in the taxonomic inference of traditional alignment-free metagenomic classifiers [14, 33]. By simplifying the Markov model based approach to taxonomic classification [16], POSMM circumvented the computational time barrier that has made several alignment-free metagenomic classifiers obsolete as the dataset size continues to grow [5]. While POSMM lacks the speed of *k*-mer aligners, it does have the speed and scalability to analyze large metagenomic datasets produced by current sequencers. As an accompaniment, POSMM offers to augment the sensitivity of faster though less sensitive alignment based metagenomic classification programs. By obviating the need for establishing model databases, made possible by generating models directly from genomic fasta files on the fly, POSMM ushers in a new approach that can be easily adapted and restructured to fit with specific needs in classification.

POSMM is also highly scalable. The memory footprint is entirely based on the size of the dataset being analyzed, as metagenomic reads are indexed and kept in memory for rapid lookup during score computation. Users with fewer resources can split datasets as needed, allowing POSMM to run on devices ranging from power-efficient laptops to high-performance computing environments. As the number of CPU cores continues to increase on desktop computers, the potential throughput of POSMM should also scale linearly. The simplified underlying codebase for generating SMMs, written in C++ 11 and only using standard libraries, is also easily portable to the increasingly common ARM architecture that continues to expand beyond use in mobile phones. The regression score models, which are built using the popular scikit library and stored in JSON, are also easily modifiable. Being able to easily adjust the score models to scale to an ever-changing and rapidly growing databases, POSMM holds the promise to remain relevant in many years to come.

## Supplementary Information

**Additional file 1**. **Table S1**: Genomes in the custom database for POSMM. **Table S2**: Final parameters for each logistic regression model. Models are taxonomic rank and model order specific, and are the result of Bayesian optimization with parameters described in the Methods section. **Table S3**: Species-level performance (Sensitivity, Precision, and F1 score), on the three simulated metagenome datasets by Kaiju, KrakenUniq, POSMM (at the confidence thresholds of 0, 0.25, 0.5, 0.75), and Kraken2 (at the confidence thresholds of 0, 0.25, 0.5, 0.75), and a hybrid of Kraken2 (at the threshold of 0) and POSMM (at the threshold of 0.25).

**Additional file 2**.** Figure S1**: Genus-level performance (F1 score), on the three simulated metagenome datasets by Kaiju, KrakenUniq, POSMM, Kraken2, and a hybrid of Kraken2 (at threshold 0) and POSMM (at threshold 0.25). A score cutoff of 0.25 was used for POSMM. For the hybrid of Kraken2 and POSMM, initial classification was obtained with Kraken2 without a cutoff, followed by genus level classification of reads left unclassified by Kraken2 with POSMM at 0.25 cutoff.** Figure S2**: Interactive (A) and static (B) sunburst diagrams of the taxonomic assignments of all reads present in the SRR062415 human saliva WGS metagenomic dataset using Kraken with no confidence score threshold.** Figure S3**: Interactive (A) and static (B) sunburst diagrams of the taxonomic assignments of all reads present in the SRR062415 human saliva WGS metagenomic dataset using POSMM at 0.25 score cutoff.** Figure S4**: Interactive (A) and static (B) sunburst diagrams of the taxonomic assignments of reads left fully unclassified by Kraken2 for the SRR062415 human saliva WGS metagenomic dataset using POSMM at 0.25 cutoff. No confidence score threshold was used for Kraken2.** Figure S5**: Interactive (A) and static (B) sunburst diagrams of the taxonomic assignments of all reads present in the SRR062462 human saliva WGS metagenomic dataset using Kraken with no confidence score threshold.** Figure S6**: Interactive (A) and static (B) sunburst diagrams of the taxonomic assignments of all reads present in the SRR062462 human saliva WGS metagenomic dataset using POSMM at 0.25 score cutoff.** Figure S7**: Interactive (A) and static (B) sunburst diagrams of the taxonomic assignments of reads left fully unclassified by Kraken2 for the SRR062462 human saliva WGS metagenomic dataset using POSMM at 0.25 cutoff. No confidence score threshold was used for Kraken2.

## Data Availability

Source codes are available at https://www.github.com/djburks/POSMM. Simulated metagenomes are available at the Kraken2 website https://ccb.jhu.edu/software/kraken/dl/accuracy.tgz.

## Abbreviations

WMS: Whole metagenome (shotgun) sequencing
ICM: Interpolated context model
POSMM: Python-optimized standard Markov model
SMM: Standard Markov model
SGM: Segmented genome model
MJSD: Markovian Jensen–Shannon Divergence
