# Supplementary material for: POSMM: an efficient alignment-free metagenomic profiler that complements alignment-based profiling
Source: Environ Microbiome. 2023 Mar 8;18:16. doi: 10.1186/s40793-023-00476-y (PMC9993663; doi:10.1186/s40793-023-00476-y)
Supplement: Supplementary file 1 — Additional file 1. Table S1: Genomes in the custom database for POSMM. Table S2: Final parameters for each logistic regression model. Models are taxonomic rank and model order specific, and are the result of Bayesian optimization with parameters described in the Methods section. Table S3: Species-level performance (Sensitivity, Precision, and F1 score), on the three simulated metagenome datasets by Kaiju, KrakenUniq, POSMM (at the confidence thresholds of 0, 0.25, 0.5, 0.75), and Kraken2 (at the confidence thresholds of 0, 0.25, 0.5, 0.75), and a hybrid of Kraken2 (at the threshold of 0) and POSMM (at the threshold of 0.25). [file 40793_2023_476_MOESM1_ESM.zip › SUPPLEMENTARY TABLES/Supplementary Table 3.docx]

Supplementary Table 3: Species-level performance (Sensitivity, Precision, and F1 score), on the three simulated metagenome datasets by Kaiju, KrakenUniq, POSMM (at the confidence thresholds of 0, 0.25, 0.5, 0.75), and Kraken2 (at the confidence thresholds of 0, 0.25, 0.5, 0.75), and a hybrid of Kraken2 (at the threshold of 0) and POSMM (at the threshold of 0.25).

|  | **Sensitivity** | **Precision** | **F1-Score** |
| --- | --- | --- | --- |
| Kraken2 HiSeq (conf. 0) | 0.478 | 0.778 | 0.592 |
| Kraken2 HiSeq (conf. 0.25) | 0.388 | 0.848 | 0.533 |
| Kraken2 HiSeq (conf. 0.5) | 0.297 | 0.91 | 0.448 |
| Kraken2 Hiseq (conf. 0.75) | 0.209 | 0.942 | 0.343 |
| Kraken2 MiSeq (conf. 0.00) | 0.401 | 0.758 | 0.524 |
| Kraken2 MiSeq (conf. 0.25) | 0.265 | 0.758 | 0.393 |
| Kraken2 MiSeq (conf. 0.5) | 0.204 | 0.794 | 0.325 |
| Kraken2 MiSeq (conf. 0.75) | 0.142 | 0.863 | 0.244 |
| Kraken2 SimBA5 (conf. 0.00) | 0.564 | 0.889 | 0.69 |
| Kraken2 SimBA5 (conf. 0.25) | 0.371 | 0.898 | 0.525 |
| Kraken2 SimBA5 (conf. 0.5) | 0.184 | 0.879 | 0.304 |
| Kraken2 SimBA5 (conf. 0.75) | 0.062 | 0.88 | 0.116 |
| KrakenUniq Hiseq | 0.467 | 0.696 | 0.559 |
| KrakenUniq MiSeq | 0.391 | 0.613 | 0.477 |
| KrakenUniq SimBA5 | 0.587 | 0.879 | 0.704 |
| Kaiju Hiseq | 0.238 | 0.802 | 0.367 |
| Kaiju MiSeq | 0.293 | 0.787 | 0.427 |
| Kaiju MiSeq | 0.341 | 0.844 | 0.486 |
| POSMM HiSeq (conf. 0) | 0.317 | 0.317 | 0.317 |
| POSMM HiSeq (conf. 0.25) | 0.319 | 0.319 | 0.319 |
| POSMM HiSeq (conf. 0.5) | 0.32 | 0.32 | 0.32 |
| POSMM HiSeq (conf. 0.75) | 0.32 | 0.32 | 0.32 |
| POSMM MiSeq (conf. 0.00) | 0.34 | 0.34 | 0.34 |
| POSMM MiSeq (conf. 0.25) | 0.332 | 0.332 | 0.332 |
| POSMM MiSeq (conf. 0.5) | 0.331 | 0.331 | 0.331 |
| POSMM MiSeq (conf. 0.75) | 0.331 | 0.331 | 0.331 |
| POSMM SimBA5 (conf. 0) | 0.555 | 0.555 | 0.555 |
| POSMM SimBA5 (conf. 0.25) | 0.555 | 0.555 | 0.555 |
| POSMM SimBA5 (conf. 0.5) | 0.555 | 0.555 | 0.555 |
| POSMM SimBA5 (conf. 0.75) | 0.555 | 0.555 | 0.555 |
| hybrid HiSeq | 0.501 | 0.707 | 0.586 |
| hybrid MiSeq | 0.501 | 0.734 | 0.595 |
| hybrid SimBA5 | 0.612 | 0.825 | 0.703 |

Conf. – Confidence Threshold
