# Supplementary figures and images for: POSMM: an efficient alignment-free metagenomic profiler that complements alignment-based profiling
Source: Environ Microbiome. 2023 Mar 8;18:16. doi: 10.1186/s40793-023-00476-y (PMC9993663; doi:10.1186/s40793-023-00476-y)

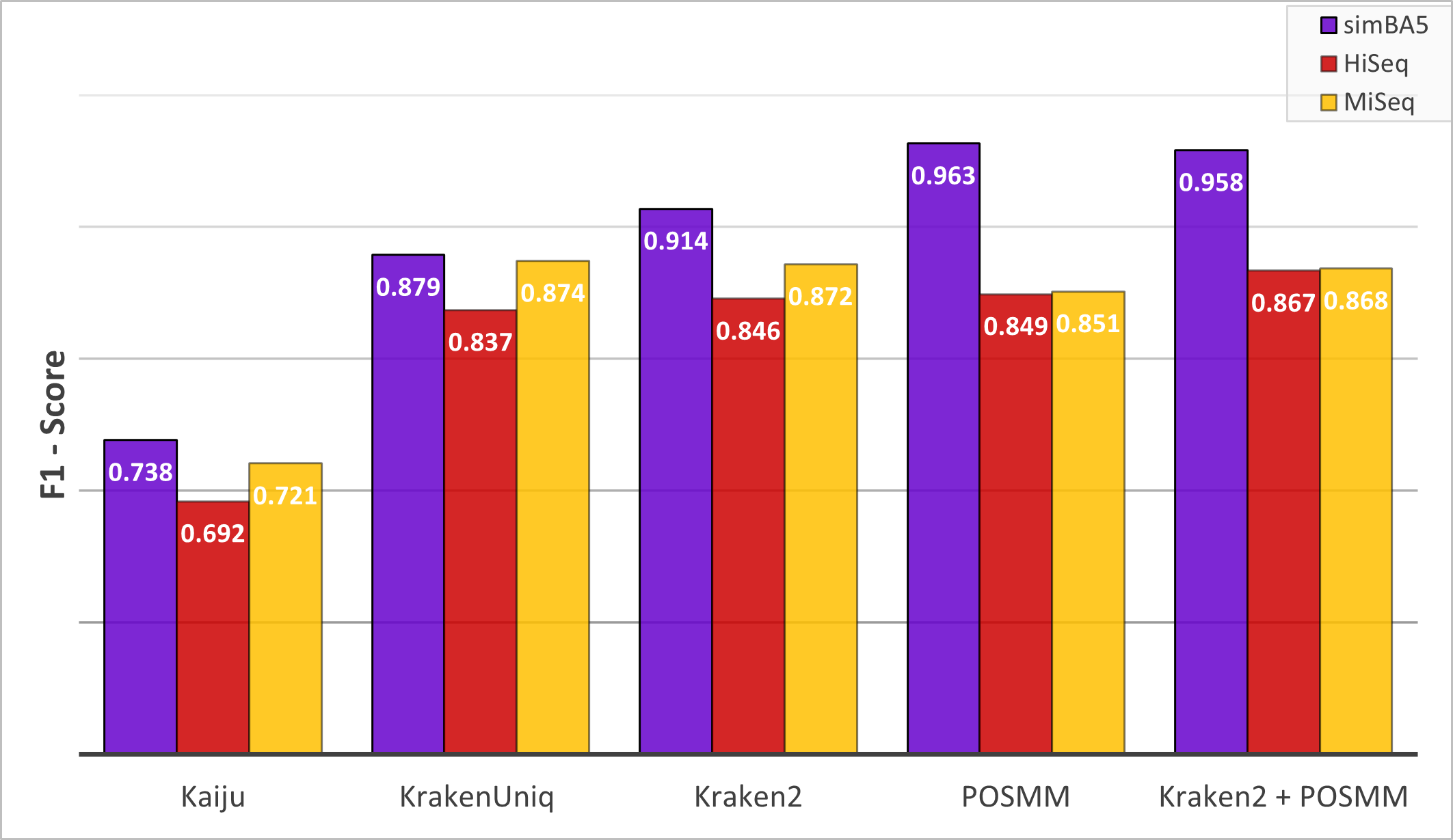

Supplement: Supplementary file 2 — Additional file 2. Figure S1: Genus-level performance (F1 score), on the three simulated metagenome datasets by Kaiju, KrakenUniq, POSMM, Kraken2, and a hybrid of Kraken2 (at threshold 0) and POSMM (at threshold 0.25). A score cutoff of 0.25 was used for POSMM. For the hybrid of Kraken2 and POSMM, initial classification was obtained with Kraken2 without a cutoff, followed by genus level classification of reads left unclassified by Kraken2 with POSMM at 0.25 cutoff. Figure S2: Interactive (A) and static (B) sunburst diagrams of the taxonomic assignments of all reads present in the SRR062415 human saliva WGS metagenomic dataset using Kraken with no confidence score threshold. Figure S3: Interactive (A) and static (B) sunburst diagrams of the taxonomic assignments of all reads present in the SRR062415 human saliva WGS metagenomic dataset using POSMM at 0.25 score cutoff. Figure S4: Interactive (A) and static (B) sunburst diagrams of the taxonomic assignments of reads left fully unclassified by Kraken2 for the SRR062415 human saliva WGS metagenomic dataset using POSMM at 0.25 cutoff. No confidence score threshold was used for Kraken2. Figure S5: Interactive (A) and static (B) sunburst diagrams of the taxonomic assignments of all reads present in the SRR062462 human saliva WGS metagenomic dataset using Kraken with no confidence score threshold. Figure S6: Interactive (A) and static (B) sunburst diagrams of the taxonomic assignments of all reads present in the SRR062462 human saliva WGS metagenomic dataset using POSMM at 0.25 score cutoff. Figure S7: Interactive (A) and static (B) sunburst diagrams of the taxonomic assignments of reads left fully unclassified by Kraken2 for the SRR062462 human saliva WGS metagenomic dataset using POSMM at 0.25 cutoff. No confidence score threshold was used for Kraken2. [file 40793_2023_476_MOESM2_ESM.zip › Supplementary Figures/Supplementary Figure 1.png]

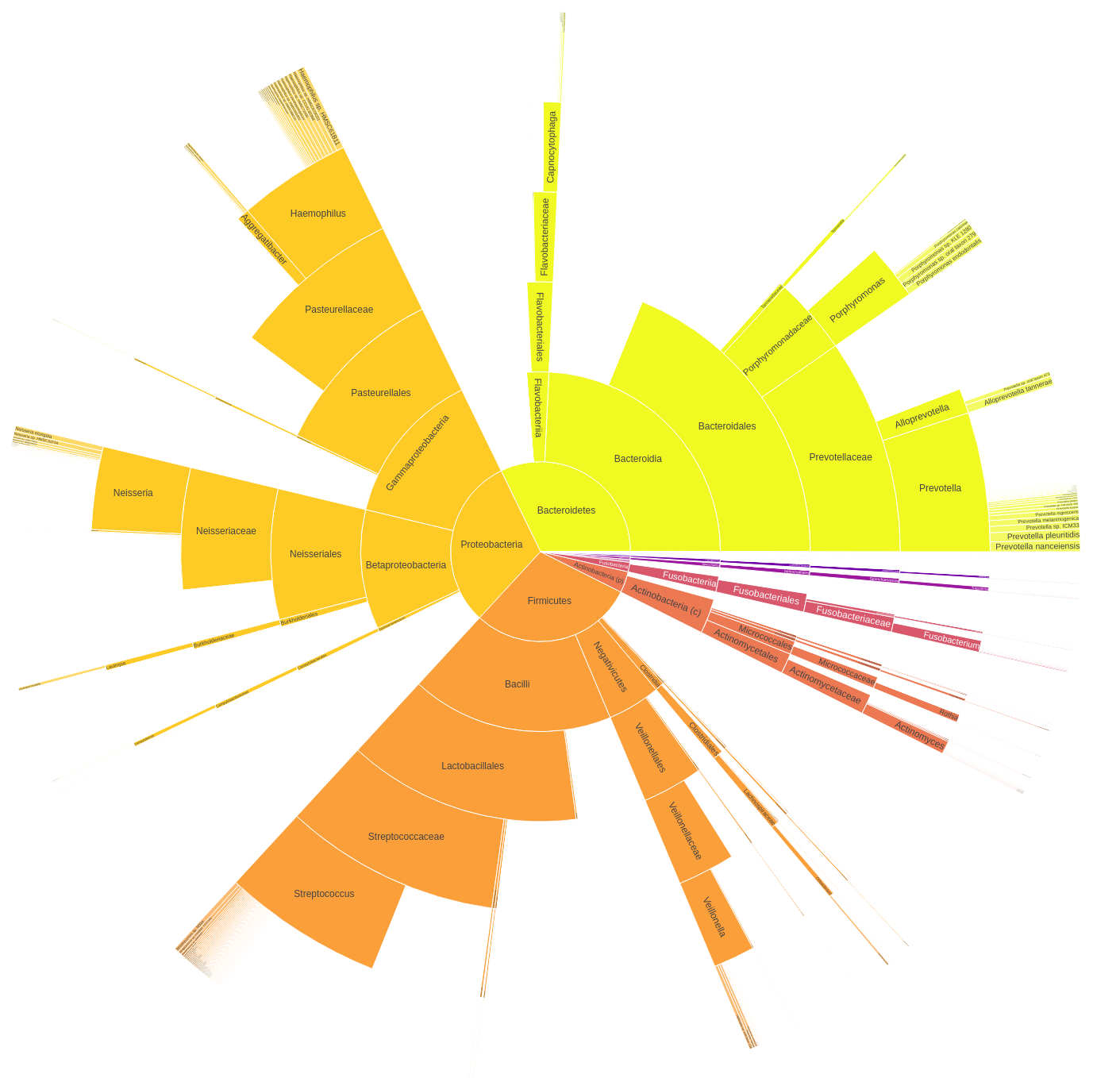

Supplement: Supplementary file 2 — Additional file 2. Figure S1: Genus-level performance (F1 score), on the three simulated metagenome datasets by Kaiju, KrakenUniq, POSMM, Kraken2, and a hybrid of Kraken2 (at threshold 0) and POSMM (at threshold 0.25). A score cutoff of 0.25 was used for POSMM. For the hybrid of Kraken2 and POSMM, initial classification was obtained with Kraken2 without a cutoff, followed by genus level classification of reads left unclassified by Kraken2 with POSMM at 0.25 cutoff. Figure S2: Interactive (A) and static (B) sunburst diagrams of the taxonomic assignments of all reads present in the SRR062415 human saliva WGS metagenomic dataset using Kraken with no confidence score threshold. Figure S3: Interactive (A) and static (B) sunburst diagrams of the taxonomic assignments of all reads present in the SRR062415 human saliva WGS metagenomic dataset using POSMM at 0.25 score cutoff. Figure S4: Interactive (A) and static (B) sunburst diagrams of the taxonomic assignments of reads left fully unclassified by Kraken2 for the SRR062415 human saliva WGS metagenomic dataset using POSMM at 0.25 cutoff. No confidence score threshold was used for Kraken2. Figure S5: Interactive (A) and static (B) sunburst diagrams of the taxonomic assignments of all reads present in the SRR062462 human saliva WGS metagenomic dataset using Kraken with no confidence score threshold. Figure S6: Interactive (A) and static (B) sunburst diagrams of the taxonomic assignments of all reads present in the SRR062462 human saliva WGS metagenomic dataset using POSMM at 0.25 score cutoff. Figure S7: Interactive (A) and static (B) sunburst diagrams of the taxonomic assignments of reads left fully unclassified by Kraken2 for the SRR062462 human saliva WGS metagenomic dataset using POSMM at 0.25 cutoff. No confidence score threshold was used for Kraken2. [file 40793_2023_476_MOESM2_ESM.zip › Supplementary Figures/Supplementary Figure 2B.png]

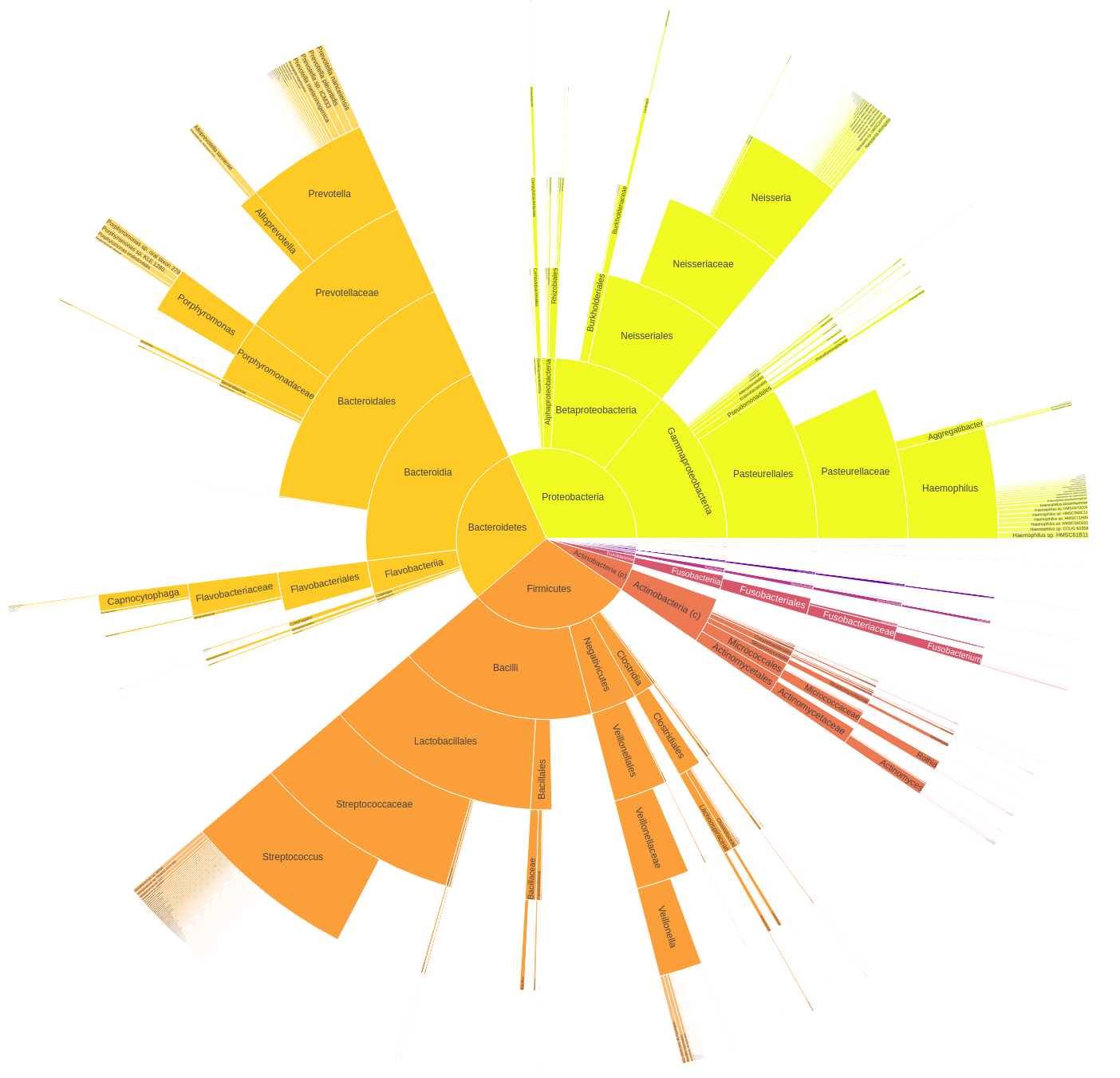

Supplement: Supplementary file 2 — Additional file 2. Figure S1: Genus-level performance (F1 score), on the three simulated metagenome datasets by Kaiju, KrakenUniq, POSMM, Kraken2, and a hybrid of Kraken2 (at threshold 0) and POSMM (at threshold 0.25). A score cutoff of 0.25 was used for POSMM. For the hybrid of Kraken2 and POSMM, initial classification was obtained with Kraken2 without a cutoff, followed by genus level classification of reads left unclassified by Kraken2 with POSMM at 0.25 cutoff. Figure S2: Interactive (A) and static (B) sunburst diagrams of the taxonomic assignments of all reads present in the SRR062415 human saliva WGS metagenomic dataset using Kraken with no confidence score threshold. Figure S3: Interactive (A) and static (B) sunburst diagrams of the taxonomic assignments of all reads present in the SRR062415 human saliva WGS metagenomic dataset using POSMM at 0.25 score cutoff. Figure S4: Interactive (A) and static (B) sunburst diagrams of the taxonomic assignments of reads left fully unclassified by Kraken2 for the SRR062415 human saliva WGS metagenomic dataset using POSMM at 0.25 cutoff. No confidence score threshold was used for Kraken2. Figure S5: Interactive (A) and static (B) sunburst diagrams of the taxonomic assignments of all reads present in the SRR062462 human saliva WGS metagenomic dataset using Kraken with no confidence score threshold. Figure S6: Interactive (A) and static (B) sunburst diagrams of the taxonomic assignments of all reads present in the SRR062462 human saliva WGS metagenomic dataset using POSMM at 0.25 score cutoff. Figure S7: Interactive (A) and static (B) sunburst diagrams of the taxonomic assignments of reads left fully unclassified by Kraken2 for the SRR062462 human saliva WGS metagenomic dataset using POSMM at 0.25 cutoff. No confidence score threshold was used for Kraken2. [file 40793_2023_476_MOESM2_ESM.zip › Supplementary Figures/Supplementary Figure 3B.png]

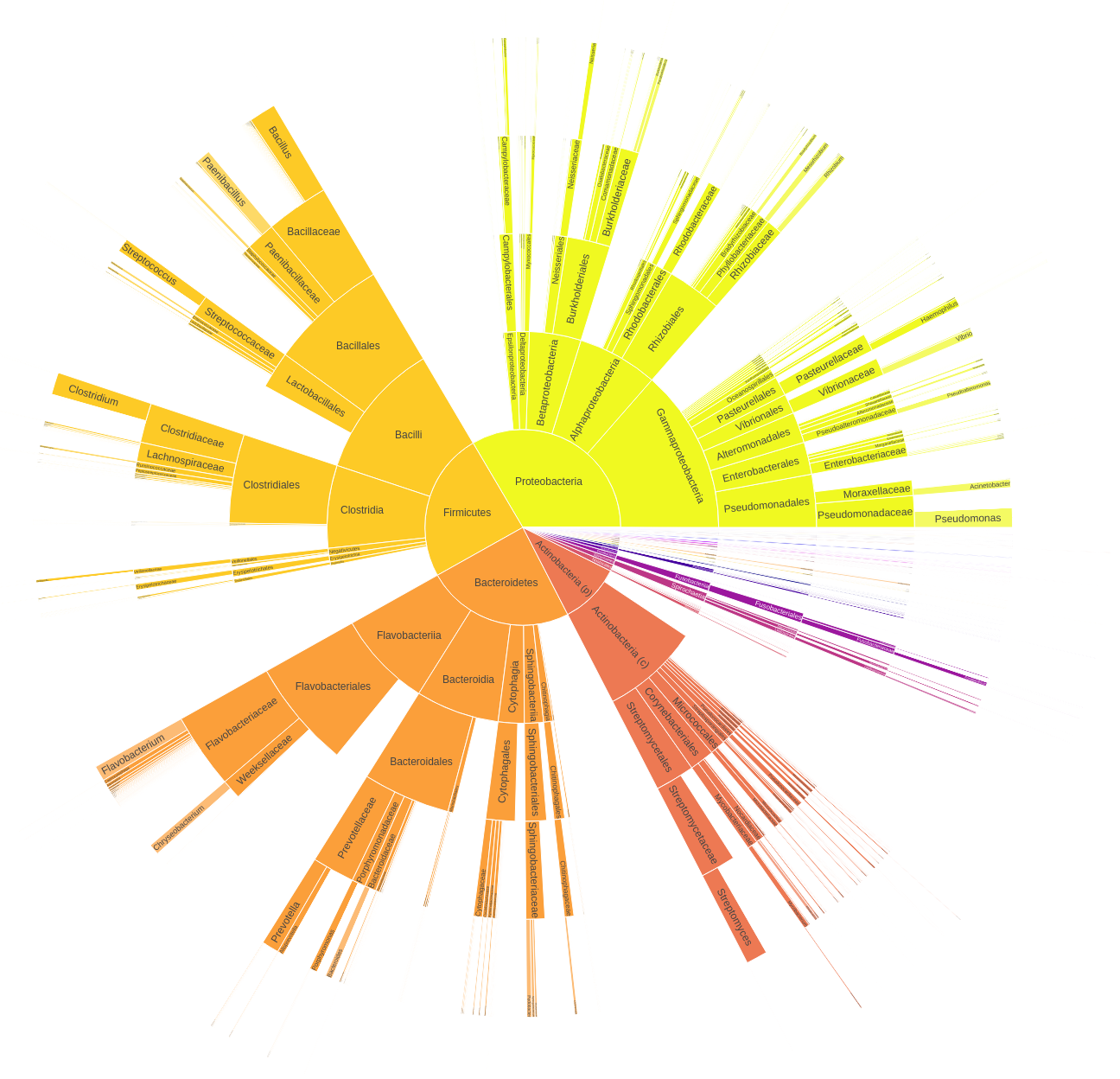

Supplement: Supplementary file 2 — Additional file 2. Figure S1: Genus-level performance (F1 score), on the three simulated metagenome datasets by Kaiju, KrakenUniq, POSMM, Kraken2, and a hybrid of Kraken2 (at threshold 0) and POSMM (at threshold 0.25). A score cutoff of 0.25 was used for POSMM. For the hybrid of Kraken2 and POSMM, initial classification was obtained with Kraken2 without a cutoff, followed by genus level classification of reads left unclassified by Kraken2 with POSMM at 0.25 cutoff. Figure S2: Interactive (A) and static (B) sunburst diagrams of the taxonomic assignments of all reads present in the SRR062415 human saliva WGS metagenomic dataset using Kraken with no confidence score threshold. Figure S3: Interactive (A) and static (B) sunburst diagrams of the taxonomic assignments of all reads present in the SRR062415 human saliva WGS metagenomic dataset using POSMM at 0.25 score cutoff. Figure S4: Interactive (A) and static (B) sunburst diagrams of the taxonomic assignments of reads left fully unclassified by Kraken2 for the SRR062415 human saliva WGS metagenomic dataset using POSMM at 0.25 cutoff. No confidence score threshold was used for Kraken2. Figure S5: Interactive (A) and static (B) sunburst diagrams of the taxonomic assignments of all reads present in the SRR062462 human saliva WGS metagenomic dataset using Kraken with no confidence score threshold. Figure S6: Interactive (A) and static (B) sunburst diagrams of the taxonomic assignments of all reads present in the SRR062462 human saliva WGS metagenomic dataset using POSMM at 0.25 score cutoff. Figure S7: Interactive (A) and static (B) sunburst diagrams of the taxonomic assignments of reads left fully unclassified by Kraken2 for the SRR062462 human saliva WGS metagenomic dataset using POSMM at 0.25 cutoff. No confidence score threshold was used for Kraken2. [file 40793_2023_476_MOESM2_ESM.zip › Supplementary Figures/Supplementary Figure 4B.png]

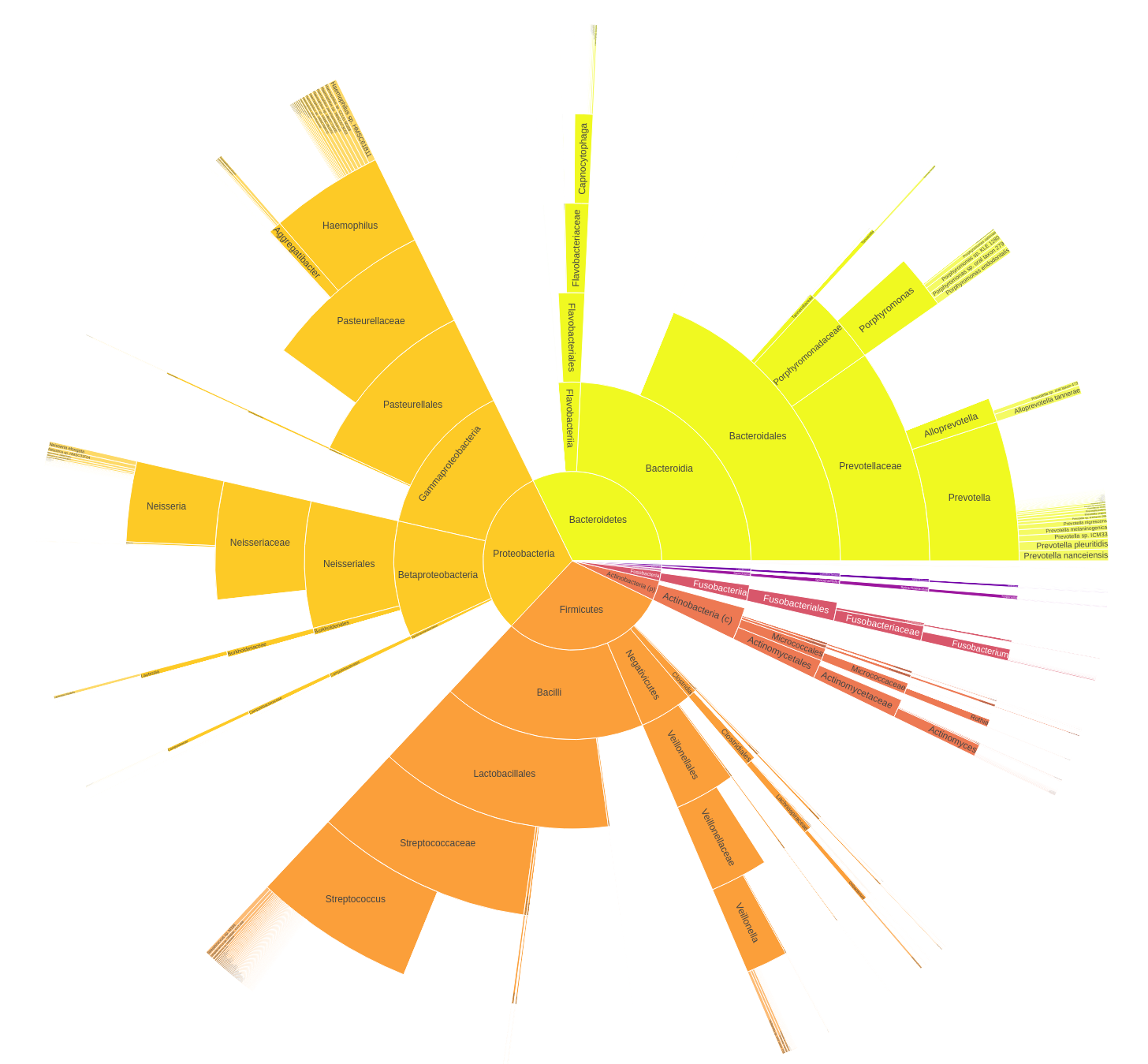

Supplement: Supplementary file 2 — Additional file 2. Figure S1: Genus-level performance (F1 score), on the three simulated metagenome datasets by Kaiju, KrakenUniq, POSMM, Kraken2, and a hybrid of Kraken2 (at threshold 0) and POSMM (at threshold 0.25). A score cutoff of 0.25 was used for POSMM. For the hybrid of Kraken2 and POSMM, initial classification was obtained with Kraken2 without a cutoff, followed by genus level classification of reads left unclassified by Kraken2 with POSMM at 0.25 cutoff. Figure S2: Interactive (A) and static (B) sunburst diagrams of the taxonomic assignments of all reads present in the SRR062415 human saliva WGS metagenomic dataset using Kraken with no confidence score threshold. Figure S3: Interactive (A) and static (B) sunburst diagrams of the taxonomic assignments of all reads present in the SRR062415 human saliva WGS metagenomic dataset using POSMM at 0.25 score cutoff. Figure S4: Interactive (A) and static (B) sunburst diagrams of the taxonomic assignments of reads left fully unclassified by Kraken2 for the SRR062415 human saliva WGS metagenomic dataset using POSMM at 0.25 cutoff. No confidence score threshold was used for Kraken2. Figure S5: Interactive (A) and static (B) sunburst diagrams of the taxonomic assignments of all reads present in the SRR062462 human saliva WGS metagenomic dataset using Kraken with no confidence score threshold. Figure S6: Interactive (A) and static (B) sunburst diagrams of the taxonomic assignments of all reads present in the SRR062462 human saliva WGS metagenomic dataset using POSMM at 0.25 score cutoff. Figure S7: Interactive (A) and static (B) sunburst diagrams of the taxonomic assignments of reads left fully unclassified by Kraken2 for the SRR062462 human saliva WGS metagenomic dataset using POSMM at 0.25 cutoff. No confidence score threshold was used for Kraken2. [file 40793_2023_476_MOESM2_ESM.zip › Supplementary Figures/Supplementary Figure 5B.png]

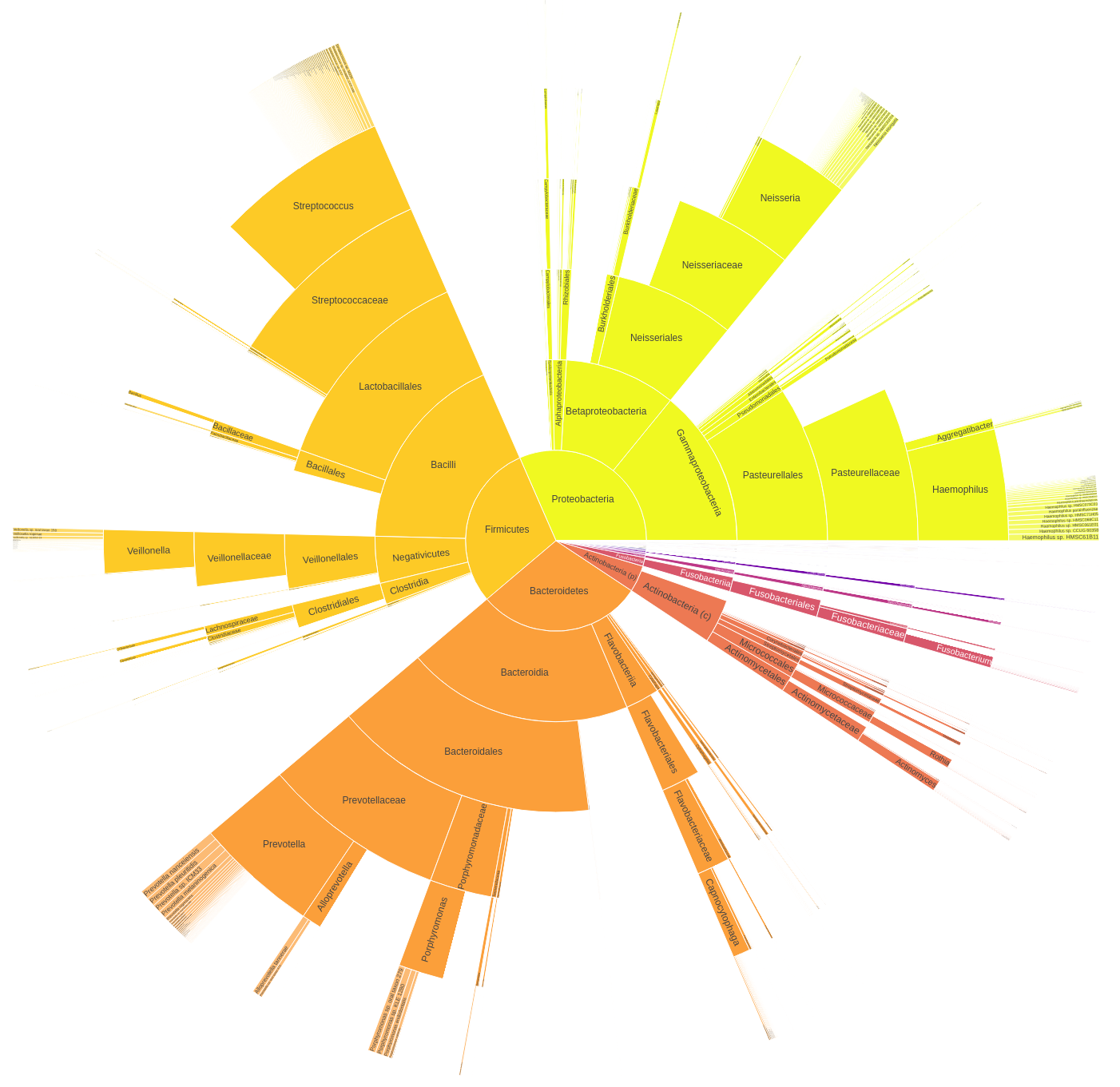

Supplement: Supplementary file 2 — Additional file 2. Figure S1: Genus-level performance (F1 score), on the three simulated metagenome datasets by Kaiju, KrakenUniq, POSMM, Kraken2, and a hybrid of Kraken2 (at threshold 0) and POSMM (at threshold 0.25). A score cutoff of 0.25 was used for POSMM. For the hybrid of Kraken2 and POSMM, initial classification was obtained with Kraken2 without a cutoff, followed by genus level classification of reads left unclassified by Kraken2 with POSMM at 0.25 cutoff. Figure S2: Interactive (A) and static (B) sunburst diagrams of the taxonomic assignments of all reads present in the SRR062415 human saliva WGS metagenomic dataset using Kraken with no confidence score threshold. Figure S3: Interactive (A) and static (B) sunburst diagrams of the taxonomic assignments of all reads present in the SRR062415 human saliva WGS metagenomic dataset using POSMM at 0.25 score cutoff. Figure S4: Interactive (A) and static (B) sunburst diagrams of the taxonomic assignments of reads left fully unclassified by Kraken2 for the SRR062415 human saliva WGS metagenomic dataset using POSMM at 0.25 cutoff. No confidence score threshold was used for Kraken2. Figure S5: Interactive (A) and static (B) sunburst diagrams of the taxonomic assignments of all reads present in the SRR062462 human saliva WGS metagenomic dataset using Kraken with no confidence score threshold. Figure S6: Interactive (A) and static (B) sunburst diagrams of the taxonomic assignments of all reads present in the SRR062462 human saliva WGS metagenomic dataset using POSMM at 0.25 score cutoff. Figure S7: Interactive (A) and static (B) sunburst diagrams of the taxonomic assignments of reads left fully unclassified by Kraken2 for the SRR062462 human saliva WGS metagenomic dataset using POSMM at 0.25 cutoff. No confidence score threshold was used for Kraken2. [file 40793_2023_476_MOESM2_ESM.zip › Supplementary Figures/Supplementary Figure 6B.png]

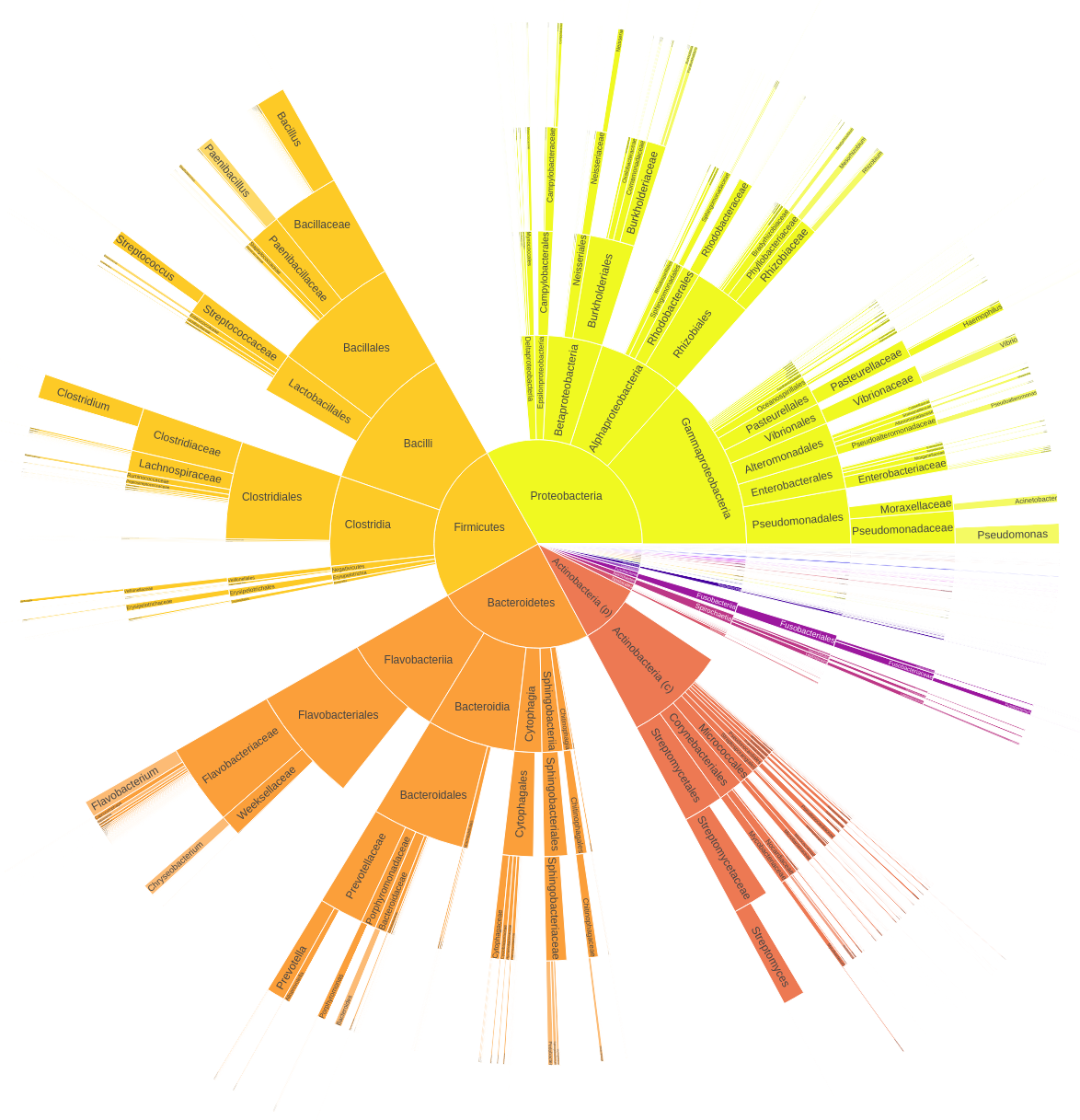

Supplement: Supplementary file 2 — Additional file 2. Figure S1: Genus-level performance (F1 score), on the three simulated metagenome datasets by Kaiju, KrakenUniq, POSMM, Kraken2, and a hybrid of Kraken2 (at threshold 0) and POSMM (at threshold 0.25). A score cutoff of 0.25 was used for POSMM. For the hybrid of Kraken2 and POSMM, initial classification was obtained with Kraken2 without a cutoff, followed by genus level classification of reads left unclassified by Kraken2 with POSMM at 0.25 cutoff. Figure S2: Interactive (A) and static (B) sunburst diagrams of the taxonomic assignments of all reads present in the SRR062415 human saliva WGS metagenomic dataset using Kraken with no confidence score threshold. Figure S3: Interactive (A) and static (B) sunburst diagrams of the taxonomic assignments of all reads present in the SRR062415 human saliva WGS metagenomic dataset using POSMM at 0.25 score cutoff. Figure S4: Interactive (A) and static (B) sunburst diagrams of the taxonomic assignments of reads left fully unclassified by Kraken2 for the SRR062415 human saliva WGS metagenomic dataset using POSMM at 0.25 cutoff. No confidence score threshold was used for Kraken2. Figure S5: Interactive (A) and static (B) sunburst diagrams of the taxonomic assignments of all reads present in the SRR062462 human saliva WGS metagenomic dataset using Kraken with no confidence score threshold. Figure S6: Interactive (A) and static (B) sunburst diagrams of the taxonomic assignments of all reads present in the SRR062462 human saliva WGS metagenomic dataset using POSMM at 0.25 score cutoff. Figure S7: Interactive (A) and static (B) sunburst diagrams of the taxonomic assignments of reads left fully unclassified by Kraken2 for the SRR062462 human saliva WGS metagenomic dataset using POSMM at 0.25 cutoff. No confidence score threshold was used for Kraken2. [file 40793_2023_476_MOESM2_ESM.zip › Supplementary Figures/Supplementary Figure 7B.png]
